# The Readability of Information and Consent Forms in Clinical Research in France

**DOI:** 10.1371/journal.pone.0010576

**Published:** 2010-05-11

**Authors:** Véronique Ménoni, Noël Lucas, Jean François Leforestier, Jérôme Dimet, François Doz, Gilles Chatellier, Jean-Marc Tréluyer, Hélène Chappuy

**Affiliations:** 1 Unité de Recherche Clinique Paris Centre, Hôpital Necker Enfants Malades, Assistance Publique Hôpitaux de Paris, Université Paris Descartes, Paris, France; 2 Laboratoire d'Ethique Médicale, Université Paris Descartes, Paris, France; 3 CIC E4 Inserm, Hôpital Européen Georges Pompidou, Assistance Publique Hôpitaux de Paris, Université Paris Descartes, Paris, France; 4 Unité de Recherche Clinique, CHD de Vendée, La Roche sur Yon, France; 5 Institut Curie, Paris, France; 6 Pharmacologie, Assistance Publique Hôpitaux de Paris, Hôpital Cochin Saint Vincent de Paul, Université Paris Descartes, Paris, France; 7 EA3620, Université Paris Descartes, Paris, France; 8 Centre d'Investigations Cliniques Paris Centre, Hôpital Necker Enfants Malades, Assistance Publique Hôpitaux de Paris, Université Paris Descartes, Paris, France; 9 Service d'Urgences Pédiatriques, Hôpital Necker Enfants Malades, Assistance Publique Hôpitaux de Paris, Université Paris Descartes, Paris, France; Children's Hospital of Eastern Ontario, Canada

## Abstract

**Background:**

Quantitative tools have been developed to evaluate the readability of written documents and have been used in several studies to evaluate information and consent forms. These studies all showed that such documents had a low level of readability. Our objective is to evaluate the readability of Information and Consent Forms (ICFs) used in clinical research.

**Methods and Findings:**

Clinical research protocols were collected from four public clinical research centers in France. Readability was evaluated based on three criteria: the presence of an illustration, the length of the text and its Flesch score. Potential effects of protocol characteristics on the length and readability of the ICFs were determined. Medical and statutory parts of the ICF form were analyzed separately. The readability of these documents was compared with that of everyday contracts, press articles, literary extracts and political speeches. We included 209 protocols and the corresponding 275 ICFs. The median length was 1304 words. Their Flesch readability scores were low (median: 24), and only about half that of selected press articles. ICF s for industrially sponsored and randomized protocols were the longest and had the highest readability scores. More than half (52%) of the text in ICFs concerned medical information, and this information was statistically (p<0.05) more readable (Flesch: 28) than statutory information (Flesch: 21).

**Conclusion:**

Regardless of the field of research, the ICFs for protocols included had poor readability scores. However, a prospective analysis of this test in French should be carried out before it is put into general use.

## Introduction

The first step in taking part in a clinical trial is, for the patient or the healthy volunteer, to express informed consent [1] [2] [3] [4]. This requires receiving and understanding potentially complex scientific information. By law, obtaining consent for research must include written information and agreement following a discussion between the physician and their patient. The written part of information, delivered as an Information and Consent Form (ICF), must be both exhaustive and understandable. Quantitative tools have been developed to evaluate the readability of written documents [5], and have been used in several studies to evaluate ICFs [6] [7] [8] [9] [10] [11] [12] [13] [14] [15] [16]. These studies all showed that such documents had a low level of readability. However, these publications were mostly aimed at specific fields of research (oncology, imaging analysis, surgery, pediatrics). We evaluated the readability of a broad range of ICFs used in clinical research protocols from various fields of research, involving diverse populations and all phases of clinical development.

## Methods

### Collection of research protocols and their ICFs

All interventional protocols involving the four public clinical research centers that received authorization by the *Comité de Protection des Personnes* (Institutional Review Board, or IRB) were included between 2001 and 2008. Protocols which did not require authorization from an IRB (data based) were excluded. The protocols conducted in these four centers include those with industrial and institutional sponsors, and most are multicenter studies generally involving other French clinical research structures. Protocols were categorized into three groups depending on their goal (therapeutic, pathophysiological or epidemiological). For each protocol, we determined: the type of sponsor (industrial or institutional), the year that the IRB granted authorization (to determine whether the protocol was submitted under the new French legislation [17] on research, resulting from the application of the European directive 2001/20/EC [18]), the field of medical research, the phase of the study, the inclusion of pediatric patients, whether or not a randomization procedure was used, whether or not the protocol was potentially life-threatening, and whether or not the protocol involved invasive tests (*i.e*. procedures, other than taking blood, which require bodily injury or which are painful). Documents not statutorily necessary and provided for those not legally responsible (minors or individuals incapable of making a decision) were also excluded. In cases where an information sheet and a consent form were given as two separate documents, the two documents were grouped together for overall analysis of the written information given to the patient. Each ICF was saved and three different analyses were conducted: one of the entire original text, one of only the paragraphs dealing with medical information and one of only the paragraphs dealing with statutory information.

### Readability determination

Three criteria were used to evaluate readability: Flesch readability score, length of the text and the presence of illustrations. The Flesch score [19] was calculated using the equation: 206.835− (1.015*sl*) − (0.846*wl*), where *sl* is the average sentence length (mean number of words per sentence) and *wl* is the average word length (mean number of syllables per word). The resulting score lies between 0, for texts that are not easily understood, and 100, for an easily understandable text. This score can be calculated with Microsoft Word® software for texts written in English, but this software is not suitable for use with French, probably due to problems with counting syllables. We therefore developed a Flesch index calculator for the French language using a PERL script, which is now freely available (http://search.cpan.org/~leforesjf/Lingua-FR-Fathom-0.01/).

### Reference texts

We also analyzed, as internal controls, a set of texts classified into four categories:

- Standard, everyday contracts (rental agreements, marriage documents, work contracts and general sales contracts for electricity, telephone and railway networks).
- Press articles taken from the two most widely distributed free newspapers (*20 minutes* and *Metro*), from the two most widely sold newspapers in France (*Le Monde* and *l'Equipe*) and from a popular medical health magazine (Santé magazine).
- Literary extracts from French authors of the 19^th^ century (Colette, Dumas, Hugo, Maupassant, Musset, Proust, Rimbaud, Stendhal and Zola).
- Politicians' speeches (by the five candidates who received most votes in the 2007 French presidential election).

### Data analysis

**Figure 1:**
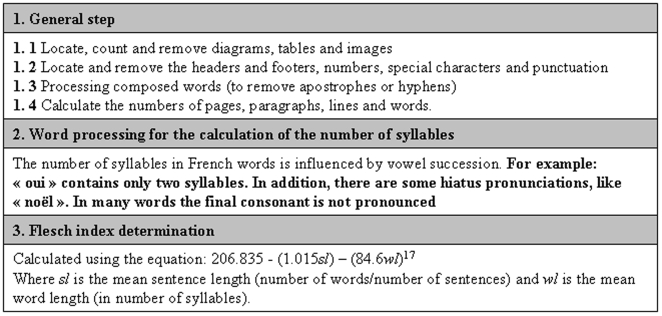
The informatic processing of information and consent documents. It includes three steps: the first is applicable to all European languages. The second step is specific to the language considered. The last step is the calculation of the Flesch index with the variables obtained from the previous steps.

Details of the protocols were entered into a Microsoft Access® database. The ICF computer files were collected and analyzed using the PERL script that we had developed (figure 1). The results obtained were entered into the database described above containing the characteristics of the corresponding protocols. Statistical analyses were performed using the NCSS® software. Results are expressed as median and 25th and 75th percentiles. For the number of words, none of the variables analyzed has a parametric distribution, so medians were compared using a Mann-Whitney test for pair-wise comparisons or a Kruskal Wallis test when more than two variables were being tested. For the Flesch scores, the variables analyzed could be compared with a t-test. Prism Graph Pad® software was used to produce graphs for comparisons between ICFs and control texts.

## Results

We included 209 protocols from 40 specialist medical fields, mainly oncology (33%), hematology (18%) and paediatrics (12%). Some protocols had several ICFs addressing different populations (adult patients, parents, guardians or proxy, such that we collected and analyzed all 275 ICFs used in these protocols. The goal of the protocols included was mostly therapeutic (65%), most were institutionally sponsored (82%) and the majority were national (74%). Sixty-six (32%) of the protocols planned to include patients under the age of 18 years.

Twenty of the 275 ICFs analyzed (7%) included a diagram or table explaining the method used in the trial or the schedule of visits. None of the everyday contracts, politicians' speeches (given orally) or the literacy extracts (only historical texts were included) contained illustrations. By contrast, all of the press articles analyzed were illustrated.

The median length of ICFs analyzed was 1304 words (991;1928), equivalent to five pages. The variables studied that had a significant influence on the length of the ICF are shown in table 1. No other factors analyzed (year of authorization by an institutional review board, the phase of the study, the inclusion of paediatric patients, or field of medical research) had an effect on ICF length. Overall, ICFs used in industrially sponsored protocols were more than twice as long as those used in institutionally sponsored protocols. Similarly, ICFs used in therapeutic protocols were one-and-a-half times longer than those used in epidemiological or psychopathological protocols. ICFs used in randomized protocols were longer than those used in open protocols. ICFS in protocols involving a risk for the patient (identified in our study as potentially life-threatening or involving invasive tests) were generally longer than those in protocols involving only minimal risks.

**Table 1:** ICF length (in number of words), Flesch scores and statistically significant differences between types and contexts of studies.

		Text length Flesch score
		N	Median	[25^e^; 75^e^]	p	Median	[25e; 75e]	p
Goal of the trial	Therapeutic	166	1571	[1055; 2499]	<0.05	24	[21; 28]	<0.05
	Epidemiological	58	1143	[890; 1354]		26	[21; 29]	
	Pathophysiological	51	1121	[900; 1309]		22	[20; 26]	
Sponsor	Institutional	244	1251	[941; 1673]	<0.05	24	[21; 28]	<0.05
	Industrial	31	2809	[1503; 4231]		26	[21; 30]	
New French legislation	Before	161	1149	[897; 1515]	<0.05	25	[21; 28]	NS
	After	114	1529	[1192; 2353]		24	[21; 27]	
Invasive tests	Yes	124	1404	[1084; 2151]	<0.05	24	[21; 28]	NS
	No	151	1273	[895; 1561]		25	[21; 28]	
Life threatening procedure	Yes	147	1450	[1048; 2245]	<0.05	24	[21; 28]	NS
	No	128	1214	[920; 1517]		25	[21; 28]	
Randomization	Yes	111	1575	[1066; 2729]	<0.05	25	[21; 29]	<0.05
	No	164	1214	[954; 1516]		24	[21; 28]	

One standard page contains around 250 words. ICF: Information and Consent Form. NS: Not statistically significant.

The median Flesch score for the entire set of ICFs was 24 (21;28). There was little variability of this score within the set of ICFs. The influence of selected factors on readability scores is shown in table 1. Factors associated with good readability were: epidemiological protocols, industrial sponsorship and use of randomization procedures. However, despite being statistically significant, the effects of these variables on the Flesch score were only weak.

Of the total content of ICFs, 52% was medical. The readability score of the medical sections was 28 (25;30), which was significantly higher (p<0.05) than the value of 21 (16;27) for the statutory sections.

**Figure 2:**
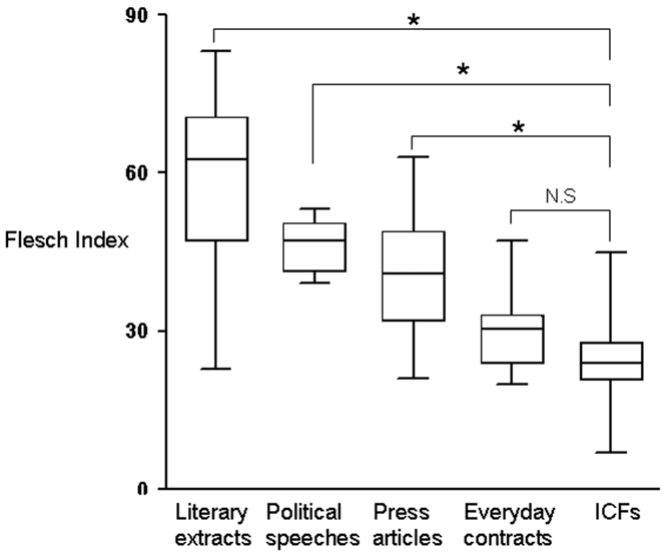
Flesch index for each category of reference texts, compared to those of information and consent forms. Central bars correspond to the medians, the ends of the boxes to the 25^th^ and 75^th^ percentiles and ends of the bars to the maximum and minimum values. ICF: Information and Consent Form. N.S: Not Significant. ICFs (N = 275) have significantly (p<0.05) lower readability scores than literary extracts (N = 9), political speeches (N = 5) and articles from the press (N = 51), but similar scores to everyday contracts (N = 6).

Comparison with the selected reference texts (figure 2) showed that the readability scores obtained for the ICFs were similar to those obtained for everyday contracts: 30 (24; 33), and significantly lower than scores obtained for literary extracts: 62 (48; 70), political speeches: 47 (41; 50) and press articles: 41(32; 50).

## Discussion

Our analysis of 209 protocols and the 275 corresponding ICFs showed a poor level of readability of these documents, as assessed using three criteria: the length of the text, the Flesch score and the presence of an illustration.

Only a small proportion of the ICFs studied included an illustration, a diagram or a table. A previous study carried out in an emergency service [20] found that patients showed a better understanding when the information documents contained illustrations. Similarly, Tait *et al.* [21] showed that patients in research trials preferred illustrated ICFs.

The Flesch scores calculated for the reference texts were highly variable, showing the sensitivity of this test: the scores varied greatly depending on the type of document analyzed. In contrast, the scores for the ICFs analyzed were mostly similar, despite the diversity of protocols included (40 different fields of medical research were represented, with both adult and pediatric protocols, and both institutional and industrial sponsorship).

The readability of the ICFs was similar to that of everyday contracts, which are renowned for their particular lack of clarity.

The Flesch scores obtained in this analysis were similar to those reported in the only other French study addressing this topic [15], in which 73 ICFs were analyzed. French ICFs were less readable than ICF written in English, where scores of between 30 and 55 were obtained [6] [7] [8] [9] [10] [12] [13] [14] [16]. Among the factors studied, we found industrial sponsorship to be associated with ICFs being both twice as long and more readable. This effect was not observed by Paris *et al*. [15]. However, they only included a small number of industrially sponsored protocols in their analysis (N = 18).

The use of randomization procedures was associated in our study with longer and more readable ICFs. The increased length of text, as previsouly reported by Paris *et al*. [15], may be because the concept of randomization needs to be described to patients, the majority of whom know very little about this procedure. The better readability obtained for ICFs for randomized protocols may thus be indicative of the efforts of the investigators to explain this concept on the ICF.

As previously shown by Mader *et al*. [13] for emergency medicine research protocols, we report that the length of the ICFs increased with protocol risk. Furthermore, we also found that the implementation of new biomedical research legislation, in 2006 [18], was associated with increased length of ICFs, but not with improved readability.

We demonstrate that the statutory sections in the ICFs were significantly less readable than the medical sections. This is despite medical information tending to contain very long words, reducing the Flesch score. This finding is consistent with a previous study [22] in which Paasche-Orlow *et al.* found a low level of readability for examples of statutory information made available to investigators by several institutional review boards.

There were two biases in our study. First, we included only those ICFs available in computer files; this allowed us to include eighty percent of all the studies conducted in the various collaborating centers. The second bias is intrinsic to the readability score used. The Flesch score evaluates a text on the basis of the length of sentences and words alone, but nevertheless allows a rapid, objective and quantitative evaluation of the complexity of a document. The use of the Flesch score to assess the readability of a text has been validated by several studies [6] [7] [8] [22]. This score itself does not reflect the level of patient understanding, because the understanding of any particular individual depends on intrinsic factors (for example, first language, culture and level of education). Nevertheless, two previous studies [21] [23] have demonstrated that patients' (adults and children) understanding of a protocol is improved when given an ICF that has a better readability. It has also been shown [20] [21] that the addition of illustrations to ICFs helps improve both understanding and acceptance of protocols by participants. However, there are no norms concerning optimal Flesch scores, numbers of words or of illustrations for ICFs; a compromise must be found between the readability of the ICF and the quantity and complexity of the information to be delivered. Identifying the best use of tools for achieving the competing goals of comprehensive and understandable information in consent forms was not among the objectives of our study. A second, interventional study is underway with this aim.

Our study showed that the readability of ICFs used in clinical research is poor, regardless of the type of protocol. The systematic use of the Flesch score may be envisaged as a way of improving readability and thus the understanding of ICFs. Certain institutional review boards have already implemented this idea [22]. However, a prospective analysis of this test in French should be carried out before it is put into general use.
